# Evaluation of the specificity of three enzyme-linked immunosorbent assays for detection of antibodies against *Salmonella* in bovine bulk milk

**DOI:** 10.1186/1751-0147-55-5

**Published:** 2013-01-30

**Authors:** Ann-Kristin J Nyman, Estelle CC Ågren, Karin Bergström, Helene Wahlström

**Affiliations:** 1 Department of Animal Health and Antimicrobial Strategies, National Veterinary Institute, Uppsala, SE-751 89, Sweden; 2 Department of Disease Control and Epidemiology, National Veterinary Institute, Uppsala, SE-751 89, Sweden

**Keywords:** *Salmonella*, Cattle, Bulk milk, ELISA, Specificity

## Abstract

**Background:**

The Swedish *Salmonella* control program has been running for decades and has resulted in a low prevalence of *Salmonella* in Swedish food producing animals. Routine bacteriology is used to detect *Salmonella*, however, bacteriology is time consuming, costly and has a low sensitivity. Different enzyme-linked immunosorbent assays (ELISAs) have been developed for detection of antibodies against *Salmonella* Dublin and *S*. Typhimurium in bovine bulk milk, individual milk samples as well as in sera. Screening bulk milk for antibodies against *Salmonella* spp. could improve the cost-effectiveness of the surveillance in Swedish dairy cattle, but as characteristics of tests may vary in different populations, tests should always be evaluated in the specific population where they will be used. Hence, the aim of this study was to evaluate the specificities of three bovine ELISAs when used to analyse bulk milk samples from Swedish dairy cattle. A second aim was to compare the performance of the two Dublin ELISAs tested.

**Methods:**

Bulk milk samples for analysis were randomly selected from samples collected within the Swedish bulk milk sampling scheme and analyzed with the three ELISAs; a Danish in-house Dublin ELISA, PrioCHECK® Salmonella Ab bovine Dublin ELISA and PrioCHECK® Salmonella Ab bovine ELISA (hereafter named mixed ELISA). The specificities of the ELISAs were calculated assuming a disease-free status in Sweden i.e. that all test positive samples were assumed to be false positive results. This assumption can be used when a disease is known to be infrequent.

**Results:**

The calculated specificities of the two Dublin ELISAs and the mixed ELISA, when using the producer’s recommended cut-off value of the corrected optic-density percent (ODC%) were 99.4% (95% Confidence Interval (CI): 98.8% -99.8%), 99.4% (95% CI: 98.8% -99.8%) and 97.9% (95% CI: 96.8% -98.7%), respectively. The correlation between the ODC% values of the two Dublin ELISAs was 0.83.

**Conclusions:**

We conclude that the evaluated ELISAs have sufficiently high specificities to be used as supplement to bacteriological examinations in the Swedish *Salmonella* control program in cattle as well as a primary screening test in routine surveillance for *S.* Dublin.

## Background

The Swedish *Salmonella* control program has been running since 1961 and has resulted in a very low prevalence of *Salmonella* infection in Swedish food producing animals [1,2]. All serotypes of *Salmonella* are encompassed by the program.

The surveillance program of Swedish cattle herds is based on required sampling of clinical suspicions of salmonellosis, routine sampling of calves submitted for post mortem examination and sampling of lymph nodes at slaughterhouses [2]. Whenever *Salmonella* is detected in a herd, the herd is put under restrictions and an eradication program is performed until two consecutive fecal samplings representing all individuals within the herd are culture negative. Closing down the sanitary slaughter during the late nineties, where animals with clinical disease were culled and sampled for *Salmonella*, resulted in reduced sensitivity of the surveillance for *Salmonella* in Swedish cattle [3]. This raised a need for new and cost-effective tools for surveillance and control of *Salmonella* in Swedish cattle.

As the consequences of a positive *Salmonella* test are extensive in Sweden, both for farmers as well as for the authorities, false positive results are undesirable. This has been avoided by use of bacteriological culture as the only analytical method within the program, as the specificity of culture is considered to be close to 100%. However, as the sensitivity of fecal culture at the individual level is low, all animals in the herd need to be sampled to obtain a herd level diagnosis. Sampling of all animals in a herd is costly and time consuming. Enzyme-linked immunosorbent assays (ELISAs) have been developed for detection of antibodies against *Salmonella* Dublin and *S.* Typhimurium in sera, individual milk samples and bulk milk [4-7]. Single bulk milk samples have been shown to have low sensitivity for detecting *Salmonella* infected herds but combinations of samples such as bulk milk and serum samples from calves or repeated bulk milk samples have been reported to achieve high sensitivity at herd level [8-10]. The specificity of bulk milk samples has been evaluated in several studies and has been shown to range from 0.95 to 1.00 depending on, among other things, underlying herd level prevalence [6,9]. As test performance may vary in different populations it is important to evaluate new serological tests in the current population before considering use within a control program.

The aim of this study was to evaluate the specificities of three ELISA tests in the Swedish dairy cattle population; a Danish in-house Dublin ELISA (hereafter named Danish Dublin ELISA) and two ELISA kits developed by Prionics AG, Switzerland (PrioCHECK® Salmonella Ab bovine Dublin ELISA, hereafter named Prionics Dublin ELISA and PrioCHECK® Salmonella Ab bovine ELISA, hereafter named Prionics mixed ELISA). Both the ELISAs developed by Prionics originate from the Danish National Veterinary Institute. A second aim was to compare the performance of Prionics Dublin ELISA with the Danish Dublin ELISA.

The present study was a part of Prionics AGs field evaluation of the Prionics Dublin and mixed ELISAs. These tests are now, after minor adjustments, sold by Prionics AG, and are presently the only commercial kits on the market for detection of *Salmonella* antibodies in cattle.

## Methods

The sample size for estimating the specificity of the diagnostic tests was calculated using the exact binomial method (as the specificity was expected to be close to 1): $\left(\begin{array}{c} n\\ x \end{array}\right)P^{x}{\left(1-P\right)}^{n-x}$; where *P* is the hypothesized proportion, *n* is the sample size, and *x* is the number of observed “successes” [11]. The tests specificities was assumed to be 99% (*P*=0.99), to have a precision of ±1%, and the estimated specificity should be within these limits with a 99% confidence interval. This required a sample size of 974.

The samples for analyses were selected from samples collected within the national bulk milk sampling scheme including samples from all Swedish dairy herds (approximate 7100 herds in 2007 [12]). The sampling was performed in the autumn of 2007. Samples from herds under restrictions were omitted as they were assumed to be infected. The first milk sample was randomly collected from the six first received milk samples of the national bulk milk sampling and thereafter every sixth milk sample was selected, to reach the calculated sample size. If sufficient material was not available in the selected milk sample, the next sample was collected. In total, bulk milk samples from 1069 different herds were collected.

The samples were stored at -20ºC in duplicates, and additionally, one sample was sent to the National Veterinary Institute DTU in Denmark for analysis using the Danish Dublin ELISA. The remaining samples were used for analysis with Prionics Dublin and Mixed ELISAs. The Danish Dublin ELISA, including O antigen O:1, 9 and 12, mainly detects antibodies against *S*. Dublin. This test was performed according to the methods of Hoorfar *et al.*[5,6] and a cut-off value of corrected optic-density percent (ODC%) ≥ 55 was used. The duplicate samples were analyzed at the National Veterinary Institute in Sweden using Prionics mixed ELISA (including O-antigens 1, 9 and 12 and 1, 4, 5 and 12, mainly detecting *S*. Dublin and *S*. Typhimurium) and Prionics Dublin ELISA (including O-antigens 1, 9 and 12, mainly detecting *S*. Dublin). The analyses were performed according to the instructions from the manufacturer (Prionics AG, Switzerland); test samples were placed in the wells of the test plates and incubated at room temperature (22±3°C). Subsequently, the plates were washed and the horseradish peroxidase conjugate was added and then the plates were incubated for 60±5 min at room temperature (22±3°C). Thereafter the plates were washed and the ready-to-use chromogen (tetramethylbenzidine) substrate was dispensed to all wells of the test plate. After incubation for 15 min at 22±3°C the colour development was stopped (using a ready-to-use stop solution) and measured at 450 nm. According to the producer’s recommendations a sample is considered positive if the ODC% (as termed percent positivity, PP-value by the producer) is ≥ 35.

### Statistics

Descriptive statistics of the ODC% were performed using distributional graphs and scatter plots. Moreover, the geographic representativeness of the collected samples was investigated, comparing the number of collected samples from different geographic areas (at that time Sweden had 8 different regional dairy livestock organizations) with the total number of dairy herds within those areas.

The specificities of the three ELISAs were calculated assuming a disease-free status in Sweden i.e. that all test positive samples were assumed to be false positive results. This assumption can be used when a disease is known to be infrequent [13].

Confidence intervals (CI) of the estimates of the specificity were calculated and graphs showing how the specificities changes with different ODC% cut-offs values were made in Stata Statistical Software (Release 11.2; College Station, TX, USA: StataCorp LP). The correlation coefficient between the ODC% values of the Danish Dublin ELISA and the Prionics Dublin ELISA, and between the Prionics ELISAs were calculated using Stata Statistical Software. Moreover, the expected number of test-positive herds was calculated, if any of the ELISAs should be used in the present Swedish dairy herd population (2011; [14]), at the producers recommended cut-off value of ODC% 35 and ODC% 55, respectively, and for comparison with a lower cut-off, ODC% 20 and 25, respectively.

## Results

Due to small amounts of milk in some of the 1069 sample tubes all samples could not be analysed with all ELISAs. The number of analysed samples with each test is presented in Table 1. The results of the geographic representativeness showed that 14-20% (mean=17%) of the herds in each region were sampled.

**Table 1:** **Specificities of three different ELISAs detecting antibodies against *Salmonella *when used in Swedish dairy cattle using the ODC% value recommended by the producer as cut-off value**

**Test**	**No. of samples**	**Specificity (95% CI**^**1**^**)**
Danish Dublin in-house ELISA (cut-off at ODC% 55)	1067	99.4% (98.8% -99.8%)
Prionics Dublin ELISA^2^ (cut-off at ODC% 35)	1065	99.4% (98.8% -99.8%)
Prionics mixed ELISA^3^ (cut-off at ODC% 35)	988	97.9% (96.8% -98.7%)

^1^CI: confidence interval.

^2^PrioCHECK® Salmonella Ab bovine Dublin ELISA.

^3^PrioCHECK® Salmonella Ab bovine ELISA.

**Figure 1:**
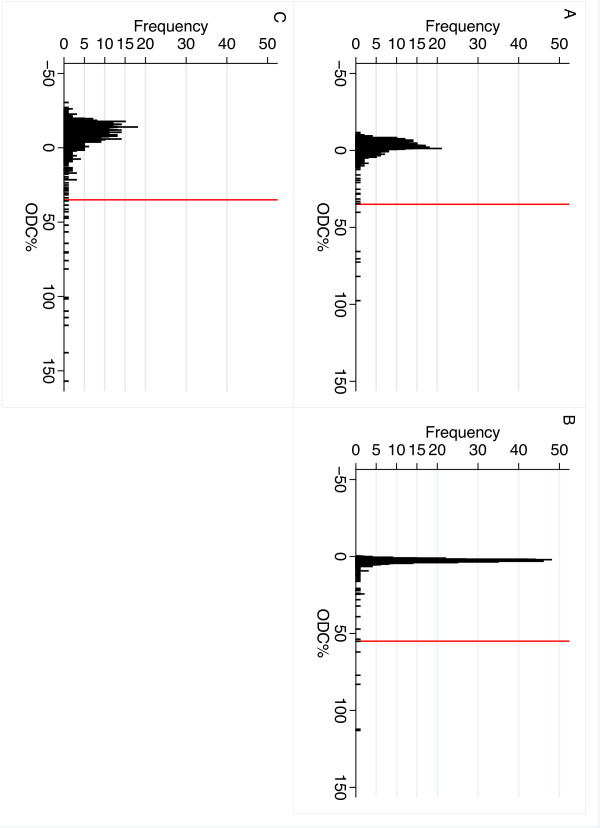
**Distribution of the corrected optic-density percent of three different ELISAs detecting antibodies against *Salmonella.*** Distributional graphs of the corrected optic-density percent (ODC%) of three different serological analyses of randomly selected bulk milk samples from Swedish dairy herds (2007). **A**: Prionics Dublin ELISA (PrioCHECK® Salmonella Ab bovine Dublin ELISA), n=1065; **B**: a Danish in-house Dublin ELISA, n=1067; **C**: Prionics mixed ELISA (PrioCHECK® Salmonella Ab bovine ELISA), n=988. The red line represents the producers recommended cut-off for these ELISAs

**Figure 2:**
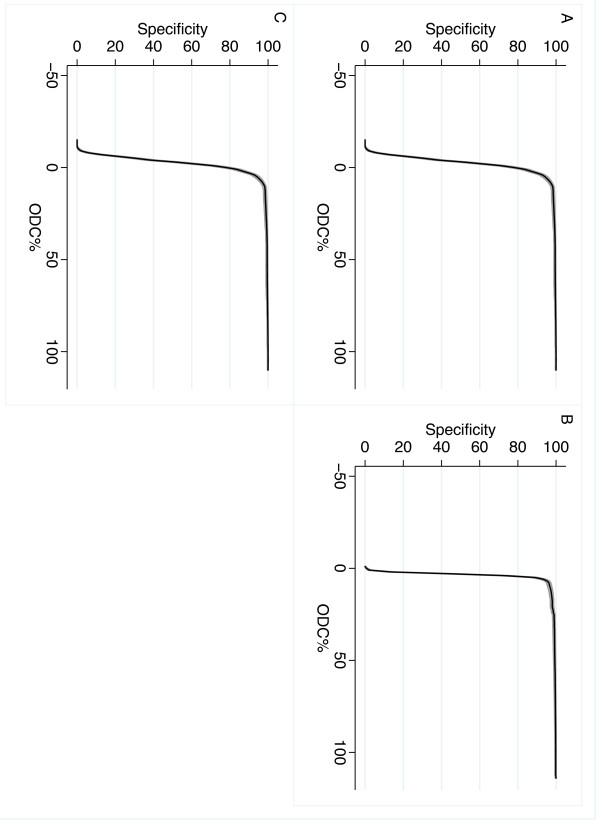
**The specificity at different cut-off values of three different ELISAs detecting antibodies against *Salmonella*.** The specificity and 95% confidence interval at different cut-off values of three different ELISAs detecting antibodies against *Salmonella*. **A**: Prionics Dublin ELISA (PrioCHECK® Salmonella Ab bovine Dublin ELISA), n=1065; **B**: a Danish in-house Dublin ELISA, n=1067; **C**: Prionics mixed ELISA (PrioCHECK® Salmonella Ab bovine ELISA), n=988. The red line represents the producer’s recommended cut-off value for these ELISAs

The distributions of ODC% value for all three ELISAs are shown in the histograms in Figure 1. The calculated specificities of the ELISAs for different ODC% cut-off values are shown in the graphs in Figure 2. The calculated specificities and 95% CI when using the producer’s recommended cut-off values are shown in Table 1. Expected numbers of test-positive herds at the cut-off values of 20, 25, 35 and 55, respectively are presented in Table 2.

**Table 2:** **Expected number of test positive Swedish dairy herds in 2011 (n=5200**[14]**) at different ODC% for three ELISAs detecting antibodies against *Salmonella***

**ELISA used**	**Expected number of test positive herds**	
	**at ODC% recommended by the producer**^**1**^	**at ODC% 20 or 25**^**2**^
Danish in-house Dublin ELISA	29 (95% CI^3^: 10-63)	58 (95% CI: 30-101)
Prionics Dublin ELISA^4^	29 (95% CI: 11-63)	63 (95% CI: 33-107)
Prionics mixed ELISA^5^	105 (95% CI: 69-168)	190 (95% CI: 138-266)

^1^ODC% 35 for Prionics ELISAs and ODC% 55 for the Danish in-house ELISA.

^2^ODC% 20 for Prionics ELISAs and ODC% 25 for the Danish in-house ELISA.

^3^CI: confidence interval.

^4^PrioCHECK® Salmonella Ab bovine Dublin ELISA.

^5^PrioCHECK® Salmonella Ab bovine ELISA.

**Figure 3:**
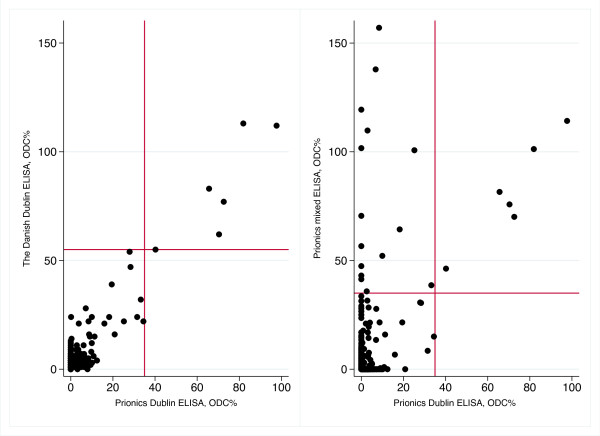
**Scatter plots comparing the results of three different ELISAs detecting antibodies against *Salmonella.*** Comparing results of serological analyses of bulk milk samples analyzed using Prionics Dublin ELISA (PrioCHECK® Salmonella Ab bovine Dublin ELISA) and a Danish in-house Dublin ELISA (n=1063), or Prionics Dublin ELISA and Prionics mixed ELISA (PrioCHECK® Salmonella Ab bovine ELISA) (n=984). The red line represents the producer’s recommended cut-off value for these ELISAs

The correlation between the ODC% values of the two Dublin ELISAs, and of the two Prionics ELISAs, was 0.83 and 0.56, respectively. Scatter plots of the ODC% values for the two Dublin ELISAs are shown in Figure 3, as well as a scatter plot of the ODC% values for the two Prionics ELISAs.

## Discussion

The specificities of the evaluated tests were high when using the ODC% cut-off value recommended by the producer. The specificity curves (Figure 2) show that the cut-off value can be decreased with the specificity remaining high for the Dublin ELISAs. For the mixed ELISA the calculated specificity was slightly less than for the Dublin ELISAs and it decreased faster when lowering the cut-off value. Our results are in agreement with Veling *et al.*[9] where the specificity of a Dublin lipopolysaccharide ELISA was 100% using bulk milk from 200 Swedish dairy herds.

The calculations used in the present study are based on the assumption that all positive results were false positives*,* i.e. an assumption that the samples originate from a disease-free population. Despite the extensive sampling within the present Swedish control program, on average only nine cattle herds per year (approximately 0.2%) have been detected positive for *Salmonella* during the last five years. Hence, we think it is reasonable to assume that the Swedish dairy population has a low prevalence of *Salmonella* and the assumption for the calculations of the tests specificities used in the present study is valid. This assumption was also used by Veling *et al.*[9] in the above mentioned study, where he used bulk milk from Swedish dairy herds from the northern part of Sweden, a region where *Salmonella* infected herds only very seldom are detected. However, as *Salmonella* infection does occur at a low prevalence we can expect that some of the positive samples are true positives. This is supported by the fact that the geographical distribution of *S.* Dublin positive cattle herds in Sweden is known to be uneven with a cluster in the south east of Sweden [15] which coincided with the geographical distribution of *S*. Dublin test positive samples in this study.

A larger number of herds tested positive with Prionics mixed ELISA than with the Dublin ELISAs. This could be explained by an expected larger number of true positive herds being detected with the mixed ELISA, rather than a lower specificity of this test. Other serotypes than *S.* Dublin are isolated from about half of the Swedish cattle herds detected with *Salmonella*, which is in line with the results of this study where the number of test positive herds with Prionics mixed ELISA were about twice as many as with the Dublin ELISAs.

Given that some of the positive samples in this study are true positive, the specificity estimates are underestimated and the true specificities of these tests are probably even higher than was shown in our study. Although the specificities of the ELISAs are high in the general Swedish dairy population, the specificities may decrease when used in subpopulations with a higher prevalence of *Salmonella* as antibodies persist after a *Salmonella* infection has been cleared from a herd [16]. In Sweden, there will be interventions in all test-positive herds and therefore it is important to evaluate the specificity of a *Salmonella* test before using it more extensively. It is also of great value for the authorities to be able to estimate the expected number of test-positive herds, in order to estimate workload and costs following for example a national bulk milk screening. Serial testing of herds, with an initial bulk-milk screening with follow up examination of positive herds with individual serology and/or bacteriology before restrictions are put on a herd, could be a cost-effective alternative. This would result in an increased number of detected herds compared to the number detected in the present control program. The expected number of test-positive herds in Sweden at present was calculated based on the results of this study at different cut-off values. Decreasing the cut-off value from ODC% 35 or 55 to 20 and 25, respectively, would roughly double the number of test-positive herds. However, decreasing the cut-off value will increase the sensitivity which is desirable. As the true *Salmonella* status of the sampled herds was not known in the present study the sensitivity of the ELISAs was not evaluated.

The correlation between the Danish Dublin ELISA and Prionics Dublin ELISA was high, which was expected as Prionics ELISA has been developed from the Danish ELISA. Moreover, the comparison between the Prionics Dublin ELISA and mixed ELISA shows the usefulness of using both ELISAs’. If the aim of an investigation is to screen for *Salmonella* in more general terms the mixed ELISA should be used, and the Dublin ELISA can be used in a serial testing to distinguish *S.* Dublin herds.

The tests used in this study are primarily designed for detecting *S.* Dublin and *S.* Typhimurium. However, other *Salmonella* serotypes can cross-react, especially in the mixed ELISA [6,17]. In Sweden, the majority of herds detected with *Salmonella* are infected with *S.* Dublin or *S.* Typhimurium, and only a small proportion of cattle herds have been detected with other serotypes. From the Swedish point of view it is an advantage if antibodies against the vast majority of *Salmonella* serotypes occurring in cattle herds can be expected to be detected by the mixed ELISA. However, the Swedish control program encompasses all serotypes of *Salmonella*, and therefore serology can be a very good complement to, but never completely substitute, bacteriology in *Salmonella* surveillance.

## Conclusions

We conclude that the evaluated ELISAs have sufficiently high specificities to be used as supplement to bacteriological examinations in the Swedish *Salmonella* control program in cattle as well as a primary screening test in routine surveillance for *S.* Dublin.

## Competing interests

The authors declare that they do not have any competing interests.

## Authors’ contributions

HW initiated and designed the study. AN was assigned as project coordinator, and EÅ and KB contributed with their expertise in epidemiology and serology/bacteriology, respectively. AN and EÅ performed all statistical calculations, and have been equally active in writing this paper. All authors were involved in the interpretation of results and drawing of conclusions. All authors have read and approved the final manuscript.

## References

[B1] Surveillance of infectious diseases in animals and humans in Sweden 2011, SVA's report series 25 2012 Uppsala, Sweden: National Veterinary Institute (SVA) 66 82 ISSN 1654-7098

[B2] Vågsholm I Viske D Review of the Swedish Salmonella control programme - road map [in Swedish] 2007 Jönköping, Sweden: Swedish Board of Agriculture 10

[B3] Wahlström H Disease surveillance in Swedish production animals [in Swedish] 2003 Sweden, Uppsala: National Veterinary Institute (SVA)

[B4] Hoorfar J Bitsch V Evaluation of an O-antigen ELISA for screening cattle herds for Salmonella typhimurium Vet Rec 1995 137 374 379 10.1136/vr.137.15.374 8578650

[B5] Hoorfar J Feld NC Schirmer AL Bitsch V Lind P Serodiagnosis of Salmonella dublin infection in Danish dairy herds using O-antigen based enzyme-linked immunosorbent assay Can J Vet Res 1994 58 268 274 7889458 PMC1263711

[B6] Hoorfar J Lind P Bitsch V Evaluation of an O antigen enzyme-linked immunosorbent assay for screening of milk samples for Salmonella dublin infection in dairy herds Can J Vet Res 1995 59 142 148 7648527 PMC1263752

[B7] Veling J Barkema HW Van der Schans J Van Zijderveld F Verhoeff J Herd-level diagnosis for Salmonella enterica subsp. enterica serovar Dublin infection in bovine dairy herds Prev Vet Med 2002 53 31 42 10.1016/S0167-5877(01)00276-8 11821135

[B8] Wedderkopp A Stroger U Bitsch V Lind P Testing of bulk tank milk for Salmonella Dublin infection in Danish dairy herds Can J Vet Res 2001 65 15 21 11227189 PMC1189636

[B9] Veling J Van Zijderveld FG Van Zijderveld-van Bemmel AM Schukken YH Barkema HW Evaluation of two enzyme-linked immunosorbent assays for detecting Salmonella enterica subsp. enterica Serovar Dublin antibodies in bulk milk Clin Diagn Lab Immunol 2001 8 1049 1055 11687438 10.1128/CDLI.8.6.1049-1055.2001 PMC96224

[B10] Warnick LD Nielsen LR Nielsen J Greiner M Simulation model estimates of test accuracy and predictive values for the Danish Salmonella surveillance program in dairy herds Prev Vet Med 2006 77 284 303 10.1016/j.prevetmed.2006.08.001 16979767

[B11] Fosgate GT Practical sample size calculations for surveillance and diagnostic investigations J Vet Diagn Invest 2009 21 3 14 10.1177/104063870902100102 19139495

[B12] Statistic Sweden (Statistiska centralbyrån (SCB) in Swedish) Yearbook of agricultural statistics 2008 including food statistics 2008 Örebro, Sweden: Statistic Sweden

[B13] Dohoo I Martin W Stryhn H Veterinary epidemiologic reserach 2010 2 Charlottetown, Prince Edward Islands, Canada: VER Inc.

[B14] Statistic Sweden (Statistiska centralbyrån (SCB) in Swedish) Yearbook of agricultural statistics 2012 including food statistics 2012 Örebro, Sweden: Statistic Sweden

[B15] Lewerin SS Skog L Frossling J Wahlstrom H Geographical distribution of salmonella infected pig, cattle and sheep herds in Sweden 1993-2010 Acta Vet Scand 2011 53 51 10.1186/1751-0147-53-51 21975258 PMC3198682

[B16] Nielsen LR Ersboll AK Factors associated with variation in bulk-tank-milk Salmonella Dublin ELISA ODC% in dairy herds Prev Vet Med 2005 68 165 179 10.1016/j.prevetmed.2004.12.006 15820114

[B17] Konrad H Smith BP Dilling GW House JK Production of Salmonella serogroup D (O9)-specific enzyme-linked immunosorbent assay antigen Am J Vet Res 1994 55 1647 1651 7887505

